# White blood cell count in birds: evaluation of a commercially available method

**DOI:** 10.1186/s12917-019-1834-8

**Published:** 2019-03-14

**Authors:** Lea Carisch, Martina Stirn, Jean Michel Hatt, Karin Federer, Regina Hofmann-Lehmann, Barbara Riond

**Affiliations:** 1 Clinical Laboratory, Vetsuisse Faculty, University of Zurich, Zurich, Switzerland; 2 Clinic for Zoo Animals, Exotic Pets, and Wildlife, Vetsuisse Faculty, University of Zurich, Zurich, Switzerland; 3 Walter Zoo AG Gossau, Neuchlen 200, 9200 Gossau, Switzerland

**Keywords:** White blood cell counting, Birds, Manual WBC counting, Avian hematology, Natt-Herricks-tic®

## Abstract

**Background:**

To conduct a hematological analysis of avian blood samples, standard automated cell counting is unreliable because all avian blood cells are nucleated. Therefore, quantitative white blood cell counting in birds is still performed manually, whereby the Natt-Herrick method is widely used in veterinary laboratories. The aim of this study was to evaluate a new commercially available single test system for avian white blood cell counting, the *Natt-Herricks-Tic®*, which would allow easy in-house analysis by clinicians or technicians. A total of 40 avian ethylenediaminetetraacetic acid (EDTA) blood samples from 24 different species were included in the study. To assess method agreement, each blood sample was analyzed for total white blood cell count with the test method and the Natt-Herrick reference method. To determine the imprecision of the reference method and the *Natt-Herricks-Tic®* method*,* the noncorrected white blood cell count was determined ten consecutive times from one avian EDTA blood sample for each method.

**Results:**

The *Natt-Herricks-Tic®* method performed well concerning staining quality and countability of the granulocytes by the hemocytometer. In the agreement study, the *Natt-Herricks-Tic®* method showed a small proportional systematic error with a small positive mean bias of 282 white blood cells/μL but had wide 95% limits of agreement (− 4683 cells/μL to 5227 cells/μL), indicating random error. The precision study resulted in a coefficient of variation of 16% for the *Natt-Herricks-Tic®* method (the mean ± standard deviation: 9.7 ×  10^3^/μL ± 1.5 × 10^3^/μL) and 23% (the mean ± standard deviation: 7.9 × 10^3^/μL ± 1.8 × 10^3^/μL) for the reference method.

**Conclusions:**

The *Natt-Herricks-Tic®* method showed acceptable precision for a manual method and demonstrated good agreement with the reference method. It can be recommended as a reliable and suitable method for determining white blood cell counts in avian EDTA blood if nonstatistical quality control measures are used in the daily routine. The application of individual reference intervals for the interpretation of white blood cell counts in birds may improve the diagnostic performance of this important analyte in a clinical setting.

## Background

Hematological analysis is an essential diagnostic part of the clinical management of avian patients to evaluate their health, clinical disease progression, and response to therapy [1]. To analyze avian blood samples, standard automated cell counting used in mammalians is unreliable because all avian blood cells are nucleated. In impedance-based hematological instruments, the nuclei of the avian erythrocytes interfere with those of white blood cells after lysis of the cells, or the lysing solutions are unable to lyse the avian blood cells adequately [2]. Therefore, quantitative white blood cell counting in birds is still performed manually with a hemocytometer.

First activities in counting white blood cells in avian blood began in 1906 by Warthin [3]. After several attempts by other authors to optimize dyes and counting procedures, Natt and Herrick refined the protocol and developed an indirect method to count avian white blood cells using a stable buffered saline solution containing methyl violet 2B, a stain commonly referred to as Natt-Herrick solution; this method has the advantages using a single dye for all cells that is stabile for a long period of two years, and the shape of the cells is retained after staining [3]. A disadvantage of Natt-Herrick solution is the difficulty to differentiate thrombocytes from lymphocytes, thus creating significant counting errors. Therefore, in the author’s Laboratory a modification of the original Natt-Herrick protocol for avian WBC counting is in use for the last 25 years, which includes the counting of only the granulocyte population in the hemocytometer, followed by a manual blood smear differential of 200 WBC and adding the lymphocyte and monocyte counts to the granulocyte counts. With this technique, avian WBC count is easier to perform and much more reliable.

Another very common quantitative counting method was the Unopette method [2, 4]. This method was based on the principle that avian eosinophils and heterophils stain with phloxine B, a red dye. However, the Eosinophil Unopette 5877 stain kit from Becton Dickinson, Rutherford, New Jersey that used this dye was removed from the market in 2007. As a replacement method, the Leukopet kit from Vetlab (Florida, USA) [5] is now available*.*

Estimation of white blood cell numbers on a stained blood smear is a technique available to most practitioners because it requires no special equipment. Thus, the average number of white blood cells evaluated in at least 10 high power fields (40 x objective) is multiplied by 2000 to obtain an estimated total white blood cell count per microliter [6]. This technique is less precise than a hemocytometer-derived white blood cell count [7].

All manual methods including the Natt-Herrick method are time consuming and require highly trained technicians [7].

To overcome some of the inherent problems of avian blood analysis and to facilitate avian white blood cell counting in a clinical setting or private practice, a commercially available, prefilled and single test system for avian white blood cell counting, the Natt-Herricks-Tic® (Bioanalytic GmbH, Umkirch, Germany) has been developed, which is evaluated in the present study. In this study, agreement between the Natt-Herricks-Tic® and a laboratory reference method (Natt-Herrick solution) was determined. Furthermore, the within-run precision for the test and the reference method were assessed, and ultimately, the practicability of the Natt-Herricks-Tic® was judged.

## Results

**Fig. 1:**
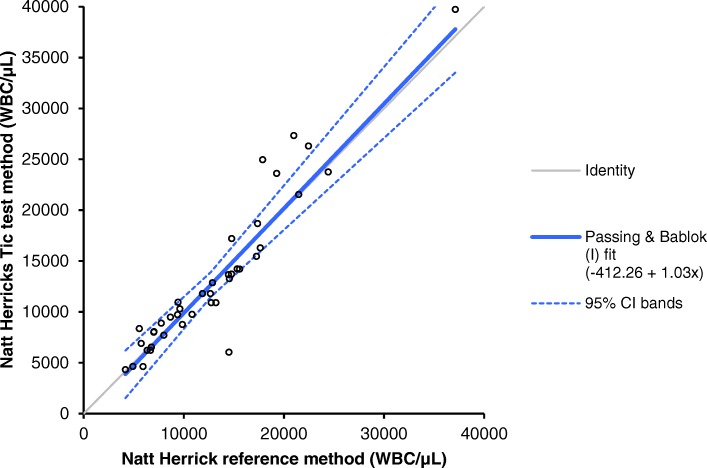
Passing-Bablok regression analysis. The thin gray line is the line of identity (y = x), and the thick blue line is the line of best fit. The blue dashed lines indicate the 95% confidence intervals

**Fig. 2:**
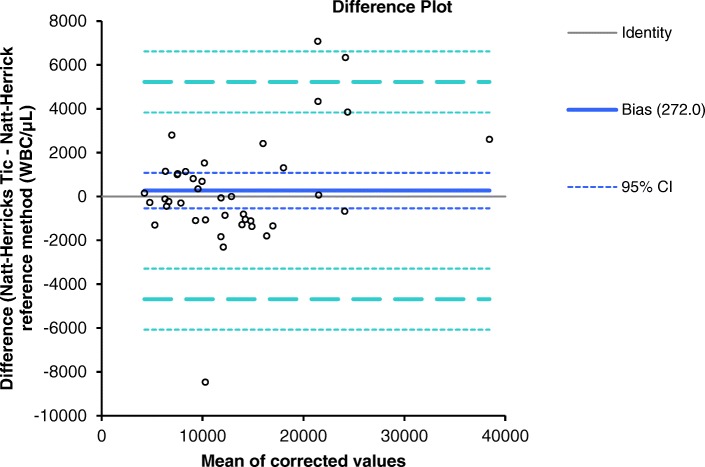
Bland-Altman difference plot for avian white blood cell count (absolute WBC/μL). The thin horizontal line (0 at the y-axis) is the line of identity; the thick blue line indicates the bias (the mean difference between the methods), with their confidence intervals as thin dashed lines. The thick dashed horizontal lines are the 95% limits of agreement with their 95% confidence intervals

Total leukocyte counts determined by the reference method ranged from 4167 cells/μL to 37,121 cells/μL. The results of the method agreement study are presented in Figs. 1 and 2. Correlation between the white blood cell counts determined with the Natt-Herrick Tic® test method and the Natt-Herrick reference method revealed a correlation coefficient of 0.95. Regression analysis by Passing-Bablok method showed very small systematic error between the test method and the reference method: intercept, 412 WBC/μL (95% confidence interval, 1.596 to 863); slope, 1.03 WBC/μL (95% confidence interval, 0.90 to 1.17) (Fig. 1). The Bland-Altman difference plot showed a very small positive bias due to systematic error (mean bias, 272 WBC/μL) but with wide limits of agreement (− 4682 to 5226) (Fig. 2), indicating random error. The Natt-Herrick Tic® test method slightly overestimated WBC counts compared to the reference method. The mean values and CV from the within-run precision study are presented in Table 1. Given that both test and reference methods are manual techniques, these CVs can be judged as good. Two samples out of 40 (5%) would have led to false clinical interpretations (Table 2). One sample from a gamefowl was falsely positive (leukopenia instead of a normal leukocyte count) with a TLC of 14,506 cells/μL with the reference method and 6035 cells/μL with the test method. A second sample from a buzzard was falsely negative (normal leukocyte count instead of leukocytosis) with a TLC of 10,823 cells/μL with the reference method and 9755 cells/μL with the test method.

**Table 1:** Within-run precision of the Natt-Herrick Tic® test method and the Natt-Herrick reference method. The results are from 10 repetitions

	Natt-Herrick Tic® method	Reference method
Mean (× 10^3^/μL)	9.7	7.9
Standard deviation (× 10^3^/μL)	1.54	1.8
Coefficient of variation (%)	16	23

**Table 2:** Number of correctly/falsely classified avian blood samples (listed according to the avian order and use of the Natt-Herrick Tic® test method for WBC counting). One of the samples was a false positive (leukopenia instead of a normal leukocyte count), and one was a false negative (normal leukocyte count instead of leukocytosis). Overall, this represented 5% of the false results (2 out of 40)

Avian order	Number of samples	Leukopenia	Normal leukocyte count	Leukocytosis	False interpretation
Psittaciformes	17	2	12	3	0
Galliformes	9	4	4	1	1 (false positive)
Accipitriformes	7	0	2	5	1 (false negative)
Anseriformes	5	2	1	1	0
Passeriformes	0	0	0	0	0
Piciformes	1	0	0	1	0
Ciconiiformes	1	0	1	0	0
Total samples	40	0	1	1	2

The Natt-Herricks-Tic® test method was easy to learn and to perform. It also performed well in terms of staining quality and countability of the granulocytes with a hemocytometer. In a few cases, recognition of the white blood cells was easier with the reference method than with the test method. However, cell counting with the hemocytometer with the 10x objective as indicated by the manufacturer is not recommended. Instead, the 40x objective was used for the agreement and precision studies for the test and reference methods.

## Discussion

For the first time, the present study evaluated a new commercially available single test system for avian white blood cell counting, the Natt-Herricks-Tic®. According to the guidelines for manual hematology of nonmammalian species published by the American Society of Veterinary Clinical Pathology (ASVCP), prior to introducing a new method as a diagnostic tool, a method validation experiment should be performed [8]. This validation procedure should be modified from standard mammalian method validation procedures to be applicable to exotic manual hematology. Thus, it should be ensured that the new method is functioning satisfactorily to meet the laboratory’s requirements and the manufacturer’s specifications [8]. To follow the ASVCP requirements, a method agreement study was performed between the test method and a reference method used for thirty years in the authors’ laboratory with a reasonable number of avian blood samples of various avian species to identify systematic errors. The results indicated that the Natt-Herricks-Tic® test method slightly overestimated white blood cell counts compared to the reference method, with a very small mean bias. However, the limits of agreement were wide, indicating that discrepancies between the measurements were due to random error. Underlying causes for this random error are most likely different staining properties of the solutions, which affected cell identification. Furthermore, the protocols for the reference and test methods showed differences in blood dilutions, and different numbers of squares in the hemocytometer were counted. Moreover, inter-observer differences most likely influenced the counting results of the present study and contributed to the wide limits of agreement. According to ASVCP guidelines, an important analytical factor is the experience and knowledge of the laboratory analysts for identifying nonmammalian white blood cells with a hemocytometer and on a blood smear [8]. In the present study, the reference method was performed by trained technicians with several years of experience in manual chamber counting and blood smear evaluation of avian blood cells, whereas the test method was performed by a Master’s student who was trained intensively in avian hematology prior to the onset of this study.

Overall, this agreement study clearly showed that bias between the two methods due to systematic error was not of significance. More importantly, random error needs to be considered a disadvantage of analytical performance. In contrast, another very small study of avian white blood cell counting comparing Natt-Herrick solution and the Unopette technique showed considerable differences between the two methods (Dein et al., 1994).

Nearly eight decades ago, manual methods used for blood cell counts in birds and reptiles were reported to be imprecise [9]. A more recent study confirmed large variations in manual counts without reporting CV [10]. For manual WBC counts, CVs were reported to range between 20 and 40% [10, 11]. In another study using Natt and Herrick solution, CVs were reported between 5.5 and 17.2% (Dein, 1994). The within-assay precision determined in the present study revealed that the CVs for the reference method and the test method were comparable to those reported in previous studies. It is very likely that the high degree of analytical imprecision contributed to the wide limits of agreement observed in the comparison study. Therefore, it is crucial to apply non-statistical quality control in daily routines by performing hemocytometer counting in duplicate, evaluating blood smears for plausibility and reducing inter-observer bias by frequent internal quality control audits of the staff.

Reference intervals are of key value in result interpretation, but interval generation is fraught with difficulties, especially concerning sample size in avian species [12]. Recommendations from the ASVCP provide allowances for sample sets of 20 or 40 samples in addition to the preferred standard of *n* ≥ 120 used to calculate reference intervals [13]. However, most published intervals for avian species do not meet the ASVCP criteria [12]; they are extremely wide and vary among species. To the best of our knowledge, lab-specific reference intervals for avian species are unavailable, and clinicians and exotics experts in zoos usually work with individual reference intervals. When interpreting the Natt-Herricks-tic® test methods results using published reference intervals for avian species, only two of 40 samples would have resulted in different clinical interpretations. Therefore, it can be concluded that the observed variations between the two methods within the present study are of minor importance, as clinical interpretation would have led to the same outcome in 95% of the cases. Clearly, this is not an assessment of diagnostic performance addressing the test methods ability to discriminate diseased and non-diseased birds. Rather, it confirms the good agreement of the test method with the reference method, considering that the diagnostic value of using published hematological reference intervals for avian patients remains questionable.

Advantageously, the test kit contains most of the consumables as prefilled sample vials filled with Natt-Herrick solution with long-term stability (2 years), end-to-end-volume capillary tubes (5 μL), and chamber filling capillary tubes. The required equipment includes a phase-contrast microscope, a hemocytometer (Neubauer improved) and a petri dish. Additional expensive pipettes are not needed. The overall handling of the test kit, ease of use, staining quality of the granulocytes and countability of the cells in the chamber were judged to be good. Counting of all leukocytes as indicated by the manufacture was not applied in this study as a clear differentiation between lymphocytes and thrombocytes was impossible. In a few cases, recognition of the granulocytes was easier with the reference method than with the test method. This may be due to different staining properties of the cells of the various avian species and to a lower degree due to differences in the Natt-Herrick solutions used for the reference method and the test method, although both method use methyl-violet 2B.

## Conclusion

The Natt-Herricks-Tic® method performed well in terms of the ease of use, staining quality and countability of the granulocytes with a hemocytometer. This method allows easy in-house analysis by clinicians or technicians. The assessment of the analytical performance of the Natt-Herricks-Tic® method showed that the test was precise enough for a manual method and demonstrated good agreement with the reference method. It can be recommended as a reliable and suitable method for determining white blood cell counts in avian EDTA blood if nonstatistical quality control measures are used in daily routines. The application of individual reference intervals for interpreting white blood cell counts in birds may improve the diagnostic performance of this important analytic tool in a clinical setting.

## Methods

EDTA blood samples from 40 birds with various clinical signs that were admitted between May 2014 and July 2015 to the Clinical Laboratory, Vetsuisse Faculty, University of Zurich, were included in the study. All samples were analyzed within 24 h after collection with the reference method and the test method. The study population consists of 22 different species: 7 Gy parrots, 2 gamefowl, 5 common buzzards, 1 capercaillie, 1 lovebird, 3 keas, 1 stork, 1 scarlet macaw, 1 bar-headed goose, 1 African pygmy goose, 1 toucan, 4 cockatoos, 1 blue-fronted amazon, 1 cockatiel, 1 domestic chicken, 1 crow, 1 Virginia quail, 1 peacock, 2 geese, 3 guinea fowl, 1 parrot, and 1 mute swan.

The within-assay precision of one EDTA-blood sample from a kea was analyzed 10 consecutive times by a skilled technician with the reference method and the Natt-Herrick-Tic® test method.

### Reference method: Natt-Herrick method [3]

Natt-Herrick solution for the reptiles and birds was prepared in-house according to the methods described by Natt and Herrick (1952). The prepared staining solution was filtered and stored at room temperature protected from light. The storage life of the staining solution is unlimited. However, filtering procedures were performed from time to time. EDTA blood samples were mixed in a ratio of 1:100 with Natt-Herrick solution. For this purpose, 990 μL staining solution and 10 μL blood were pipetted and mixed with a micropipette (Calibra, Socorex, Ecublens, Switzerland) in a sample vial (tubes 3.5 mL, Sarsted AG & Co., Nümbrecht, Germany). The sample-stain mixture was incubated and mixed for 5–10 min on an automated mixer (Rock ‘n Roller 34,201, Snijders Scientific B.V., AR Tilburg, Nederland) at room temperature. Afterwards, the sample-stain mixture was filled into a Neubauer Improved hemocytometer (BLAUBRAND®-Zählkammern, Brand Gmbh, Wertheim, Germany). The hemocytometer was incubated in a petri dish containing wet filter paper for 2 min, allowing sedimentation of the cells. After sedimentation, instead of counting all leukocytes, only the granulocytes were enumerated using a Laborlux S microscope (Leitz, Lenzburg, Germany) by counting the cells in the four large outer squares containing sixteen small squares at 400 x magnification. Counting was performed in duplicate by counting both chamber grids of one chamber. The mean of both chamber grids was calculated and multiplied by a factor of 250 to obtain the noncorrected white blood cell count (NCLC) per μL. The NCLC only includes eosinophils, basophils and heterophils. To include lymphocytes and monocytes in the total white blood cell count (TLC), the NCLC was multiplied by 100 and divided by the sum of the percentages of heterophils, eosinophils and basophils determined by microscopic differentiation.

### Test method: Natt-Herricks-tic® (bioanalytic GmbH, Freiburg, Germany)

A 5-μL end-to-end capillary tube was filled with EDTA blood and transferred into a test vial prefilled with Natt-Herricks-Tic® reagent, as provided by the manufacturer. The vial was mixed carefully until the blood was removed completely from the capillary. The sample-stain mixture was incubated and mixed for 2 to 5 min on an automated mixer at room temperature and then transferred into the Neubauer improved counting chamber using a capillary tube. The counting chamber was placed in a petri dish containing wet filter paper for 2 min, allowing sedimentation of the cells. Differently from the manufactures instruction, not all leukocytes but only granulocytes (heterophils, eosinophils, and basophils) were counted in one whole counting grid (9 large squares) of the counting chamber with the same microscope used for counting in the Natt-Herrick method at 100 x magnification (10x objective). To obtain the NCLC per μL, the number of counted cells was multiplied by a factor of 222.2. To include lymphocytes and monocytes in the TLC, the NCLC was multiplied by 100 and divided by the sum of the percentages of heterophils, eosinophils and basophils determined by microscopic differentiation.

### Differentiation of white blood cells

From each EDTA blood sample, two blood smears were prepared and stained with an automated staining instrument (HEMA-TEK 2000 slide stainer, Bayer HealthCare AG, Berlin, Germany), using a modified Wright-Giemsa solution (Hematek® Stain Pak, Siemens Healthcare Inc., New York, USA). Two technicians with experienced in avian hematology differentiated one hundred white blood cells each. Out of the two differential counts, the mean was calculated to obtain a percentage for each cell type. If deviations between the two technicians exceeded 10% for heterophils and lymphocytes, a clinical pathologist was consulted for a final decision. Finally, the percentages were used to calculate the TLC as described above.

### Quality control

Statistical quality control is inadequate to control manual white blood cell counts in birds, and standardized control blood is not available for avian species. Nevertheless, to ensure the stability of both methods, the reference and test methods, non-statistical measures of quality control were applied to each single analysis. For each sample, chamber counting was performed in duplicate, and the mean was used to calculate the NCLC. If the deviation between the two counts exceeded 15%, the counting procedure was repeated. Furthermore, correlation with a blood smear evaluation was performed to ensure plausibility of the counting results.

### Clinical relevance

To determine the clinical relevance of the observed differences between the reference and test methods for each sample, the results from the Natt-Herricks-Tic® method and the reference method (Natt-Herrick method) were compared with avian reference intervals from the current literature [14–20]. The results were classified as within, below (leukopenia) or above (leukocytosis) the reference interval. The resulting interpretations from the Natt-Herricks-Tic® and reference methods were compiled and compared to each other. If incongruous results were observed, i.e., a pathological finding in the test method (Natt-Herricks-Tic®) and not in the standard method (Natt-Herrick method), they were classified as false positive results. If no pathological finding was found in the test method (Natt-Herricks-Tic®) and a pathological finding was reported in the standard method, the result of the test method was classified as a false negative result. After completing the classification, the total number of false samples was identified, and the percentage of false samples in relation to the total number of samples was calculated.

All results were compiled in a table calculation program, and the Microsoft Excel add-in “Analyse-it” was used for statistical analyses (Analyse-it for Microsoft Excel 2010, Analyse-it-Software, Ltd. http://www.analyse-it.com). For the agreement study, TLC was used to calculate the Pearson coefficient of correlation (*r*), a Passing–Bablok linear regression analysis was performed providing an intercept and slope with a 95% confidence interval, and a Bland–Altman difference plot with biases and 95% limits of agreement was graphed. For precision analysis, the standard deviation (SD) and coefficient of variation (CV) were calculated.

## Abbreviations

ASVCP: American Society of Veterinary Clinical Pathology
CV: Coefficient of variation
EDTA: Ethylene-diamine-tetra-acetic acid
SD: Standard deviation
WBC: White blood cell
